# Clinical Associations of Osteoporosis in BCR::ABL1 Negative Myeloproliferative Neoplasms

**DOI:** 10.1155/ije/6041016

**Published:** 2026-05-16

**Authors:** Mürüvvet Seda Aydin, Simten Dagdas, Mehmet Sezgin Pepeler, Gülten Korkmaz, Aysel Alper, Elif Açıkgöz, Tugce Nur Bektas, Merve Pamukçuoğlu, Funda Ceran, Gulsum Ozet

**Affiliations:** ^1^ Department of Hematology and Bone Marrow Transplantation, Ankara Bilkent City Hospital, Ankara, Turkey; ^2^ Department of Obstetrics and Gynecology, Mersin State Hospital, Mersin, Turkey, mersindevlethastanesi.gov.tr; ^3^ Department of Anesthesiology and Reanimation, Ondokuz Mayıs University, Samsun, Turkey, omu.edu.tr; ^4^ General Practitioner, Nallihan State Hospital, Ankara, Turkey

**Keywords:** BCR::ABL1-negative myeloproliferative neoplasm, osteopenia, osteoporosis

## Abstract

**Background/Aim:**

Osteoporosis can be seen in some hematologic diseases, such as BCR::ABL1‐negative myeloproliferative neoplasms. The incidence and prevalence of osteoporosis and osteopenia in BCR::ABL1‐negative MPN are not very well known.

**Materials and Methods:**

Patients diagnosed with one of the classical BCR::ABL1‐negative myeloproliferative neoplasms, such as essential thrombocythemia, polycythemia vera, and myelofibrosis, were included in the study. Due to the retrospective design, a matched control group was not available, and comparisons with the general population were made for contextual interpretation.

**Results:**

Ninety‐four patients were included. Fifty‐eight (61.7%) patients had osteopenia, and 16 (17%) patients had osteoporosis according to femoral dual energy X‐ray absorptiometry (DXA) measurements. Fifty‐three (58.9%) patients had osteopenia, and 27 (30%) patients had osteoporosis according to lumbar DXA measurements. When both femur and lumbar DXA measurements were evaluated together, 67 (71.3%) patients had osteopenia and 31 (33%) patients had osteoporosis. In patients over 50 years of age, these frequencies were found to be 71.4% for osteopenia and 37.7% for osteoporosis. Osteopenia was significantly associated with female sex, cytoreduction, and longer disease duration (*p* < 0.05). In the multivariate analysis, female sex was found to be an independent risk factor for osteopenia (*p* = 0.009, OR: 3.70, 95% CI: 1.394–9.833). Osteoporosis was significantly associated with disease subtype, female sex, age, and no antiaggregant use (*p* < 0.05). In the multivariate analysis, older age (*p* = 0.044, OR: 1.04, 95% CI: 1.001–1.083) was found to be an independent risk factor for osteoporosis.

**Conclusion:**

Patients with BCR::ABL1‐negative classical myeloproliferative neoplasms have a higher risk of osteopenia/osteoporosis compared to the general population. DXA measurements should be performed, especially in female patients and in patients of advanced age.

## 1. Introduction

Osteoporosis is a progressive systemic skeletal disease characterized by low bone mass and microarchitectural deterioration of bone tissue, which in turn increases bone fragility and susceptibility to fractures [1]. Osteoporosis is a significant health problem worldwide, as well as in Turkiye. Fifty percent of individuals over age 50 in Turkiye have osteopenia, also 25% have osteoporosis. Secondary osteoporosis is defined as osteoporosis that occurs in association with various diseases or drug use. Secondary osteoporosis is seen in some hematological diseases such as hemophilia, leukemias and lymphomas, monoclonal gammopathies, multiple myeloma, sickle cell anemia, systemic mastocytosis, and thalassemia [2].

BCR::ABL1‐negative myeloproliferative neoplasms (MPNs), (essential thrombocythemia [ET], polycythemia vera [PV], and myelofibrosis [MF]) are among the hematologic diseases that may be associated with osteoporosis. The median ages at diagnosis of ET, PV, and primary MF are 50, 65, and 67 years, respectively [3]. Therefore, this population has a higher risk of osteopenia and osteoporosis. However, the true prevalence, related mechanisms, and therapeutic implications are not well defined in this population [4]. Moreover, the effect of disease symptom burden, together with known risk factors (age, smoking, and weight), on the development of osteoporosis is unclear. In this study, we aimed to determine the prevalence and clinical associations of osteopenia and osteoporosis in classical BCR::ABL1‐negative MPNs.

## 2. Materials and Methods

### 2.1. Patients

Patients diagnosed with one of the classical BCR::ABL1‐negative MPNs (ET, PV, and MF including primary or post‐ET/PV forms) who were admitted to the hematology unit of Ankara Bilkent City Hospital between 21 January 2019 and 01 January 2025 were included in the study. Diagnoses were established according to the World Health Organization (WHO) 2022 criteria [5]. Due to the retrospective design, a matched control group was not available. There were no predefined clinical exclusion criteria. Patients without available bone mineral density measurements were not included in the study. All patients were evaluated during routine outpatient follow‐up visits.

### 2.2. Definition

Demographic and laboratory findings such as age, sex, nationality, pregnancy and menstrual histories, comorbidities (including hemoglobinopathy), medications used, body mass index, smoking, alcohol habits, and disease duration form diagnosis were recorded; 25‐hydroxy Vitamin D, intact parathyroid hormone (iPTH), and ferritin levels were examined. Menarche after age of 15 was considered delayed menarche, menopause before age of 40 was considered premature menopause, and parity more than one was considered multiparity. Detailed medication histories were recorded for all patients. Medications not directly and consistently associated with bone metabolism or bone mineral density in the literature (such as antidiabetic or antihypertensive agents) were not included in statistical analyses due to heterogeneity of drug classes and limited sample size.

MPN‐10 symptom scores [6] were used to define the symptom burden of the patients. MPN‐10 symptom scores included questions focused on fatigue, concentration, early satiety, inactivity, night sweats, itching, bone pain, abdominal discomfort, weight loss, and fever.

Bone mineral density measurements were calculated with dual energy X‐ray absorptiometry (DXA) via T‐scores and assessments were not performed during acute hospitalizations. T‐score measurements were calculated from lumbar (L1, L1–L4) and femoral (femur neck and femur total) regions. Osteoporosis and osteopenia are defined as, respectively, a T‐score of < −2.5 and a T‐score between −1.0 and −2.5 [7].

### 2.3. Data Collection and Statistics

Data were obtained retrospectively from electronic and written records. To ensure data accuracy, inconsistencies were promptly addressed, and a feedback mechanism was implemented during data recording and analysis. Statistical analyses were performed using the Statistical Package for the Social Sciences (SPSS) Version 20.0 (SPSS Inc., Chicago, IL). Descriptive analyses were presented using medians (range) for non‐normally distributed and ordinal data. Variables potentially affecting bone mineral density such as body mass index and medications, including antiosteoporotic treatments, 25‐hydroxy Vitamin D levels, and iPTH levels were evaluated in univariate analyses. Univariate analyses were performed using the Chi‐square, Fisher’s exact, and Mann–Whitney *U* tests, where appropriate. For multivariate analysis (MVA), the possible factors identified by univariate analyses were further entered into logistic regression analysis. Receiver operating characteristic curve analysis was used to determine optimal cutoff values for continuous variables. Area under the curve (AUC) values with 95% confidence intervals were calculated. A one‐sample proportion test was performed to compare the observed prevalence of osteoporosis and osteopenia with the reported national prevalence rates. A *p* value of less than 0.05 was considered statistically significant.

### 2.4. Ethical Approval

The study was conducted ethically, and all procedures were conducted in accordance with the requirements of the World Medical Association’s Declaration of Helsinki. This study was reviewed and approved by the Institutional Review Board of Ankara Bilkent City Hospital (approval number: TABED 1‐25‐1011; approval date: 12 February 2025). Informed consent was not required because this study included retrospective observational data.

## 3. Results

### 3.1. Demographic and Clinical Variables

Ninety‐four patients with a median age of 62.9 years (range, 22.1–90.8) were included in the study. The median age at diagnosis was 57.2 years (range, 18.6–88.4). The majority of the patients were female (*n* = 55). Comorbidities in this study population were cardiovascular (63.8%), endocrine (45.7%), neurological (19.1%), pulmonary (12.8%), renal (10.6%), and liver disease (2.1%). Nine patients had a history of thrombosis. Hemoglobinopathies were detected in 5 (5.3%) patients (thalassemia trait in 3, HbD in 1). Eight (8.5%) patients (bisphosphonates in 6 and denosumab in 2 patients) were currently receiving treatment for osteoporosis. There were no patients using hormonal therapy or corticosteroids. Cytoreductive therapy (hydroxyurea, anagrelide, or ruxolitinib) was administered to 69 patients (73.4%). The proportion of patients receiving cytoreduction was 78.6% (33/42) in the ET subgroup and did not significantly differ compared with PV and MF subtypes (*p* = 0.308). The median score of MPN‐10 symptom scoring in 87 patients was 13 (range, 0–57). Serum 25‐Hydroxyvitamin D levels were available in 74 patients. None of the patients had primary hyperparathyroidism, but iPTH levels were above the upper limit of normal (> 65 ng/L) in 13 (52%) of the 25 patients with available measurements. While elevated iPTH levels suggested secondary hyperparathyroidism related to Vitamin D insufficiency in 11 patients, the cause was unknown in the remaining two patients. While 45 patients (47.9%) had depleted iron stores (ferritin < 30 μg/L), 12 patients (12.8%) had adequate iron stores (ferritin > 100 μg/L). The baseline demographic, clinical, and laboratory characteristics of the study population are presented in Table 1.

**TABLE 1:** Baseline characteristics of the study population.

Variable	Value
Age, years—median (range)	62.9 (22.1–90.8)
Age at diagnosis, years—median (range)	57.2 (18.6–88.4)
Disease duration, years—median (range)	4.09 (0.07–24.34)
Female sex, *n* (%)	55 (58.5)
Disease subtype, *n* (%)	
Polycythemia vera	34 (36.2)
Essential thrombocythemia	42 (44.7)
Myelofibrosis	18 (19.1)
BMI > 24.9 (overweight/obese), *n* (%)	70 (76.1)
Hemoglobinopathy, *n* (%)	5 (5.3)
Smoking (current/former), *n* (%)	18 (40)
Vitamin D status, *n* (%)	
Deficiency (< 20 μg/L)	50/74 (67.6)
Insufficiency (< 30 μg/L)	63/74 (85.1)
Elevated iPTH (> 65 ng/L), *n* (%)	13/25 (52)
Depleted iron stores, *n* (%)	45 (47.9)
MPN‐10 score—median (range)	13 (0–57)
Medications, *n* (%)	
Cytoreductive therapy	69 (73.4)
Antiaggregant	70 (74.5)
DOAC	8 (8.5)
Warfarin	2 (2.1)
LMWH	1 (1.1)

Abbreviations: BMI, body mass index; DOAC, direct acting oral anticoagulant; iPTH, intact parathyroid hormone; LMWH, low molecular weight heparin; MPN, myeloproliferative neoplasm.

Femoral DXA measurements were available in all patients, and lumbar DXA measurements were available in 90 patients. According to femur DXA measurements, 58 (61.7%) patients had osteopenia and 16 (17%) patients had osteoporosis. According to lumbar DXA measurements, 53 (58.9%) patients had osteopenia, and 27 (30%) patients had osteoporosis. When both femur and lumbar DXA measurements were evaluated together, 67 (71.3%) patients had osteopenia and 31 (33%) patients had osteoporosis. In patients over 50 years of age, these frequencies were 71.4% for osteopenia and 37.7% for osteoporosis. The prevalence of osteopenia in patients aged over 50 years was significantly higher than the reported national prevalence of 50% (*z* = 3.75, *p* = 0.0002; 95% CI: 59.97–81.1). Similarly, the prevalence of osteoporosis in this age group was significantly higher than the reported national prevalence of 25% (*z* = 2.57, *p* = 0.010; 95% CI: 26.9–49.4).

### 3.2. Relationship of Osteopenia and Osteoporosis With Other Variables

Osteopenia was significantly associated with female sex, longer disease duration, and cytoreduction (*p* = 0.002, *p* = 0.032, and, *p* = 0.049 respectively). No statistical difference was found for osteopenia in those using bisphosphonates or denosumab (*p* = 0.100). In female patients; delayed menarche, premature menopause, and multiparity were not found to be risk factors for osteopenia (*p* = 0.456, *p* = 1.000, and *p* = 0.320, respectively).

Osteoporosis was significantly associated with disease subtype (ET), female sex, no antiaggregant use, and higher current age (*p* = 0.023, *p* = 0.030, *p* = 0.011, and *p* = 0.044, respectively). Among the eight patients with a history of bisphosphonate or denosumab use, six had DXA measurement consistent with osteoporosis, while two patients had nonosteoporotic DXA measurements (*p* = 0.014). In female patients; delayed menarche, premature menopause, and multiparity were not found to be risk factors for osteoporosis (*p* = 1.000, *p* = 1.000, and *p* = 0.224, respectively).

In a univariate analysis, femoral osteoporosis was significantly associated with higher current age (*p* = 0.004), higher age at diagnosis (*p* = 0.014), the ET subtype (*p* = 0.007), lower BMI (*p* = 0.001), no direct‐acting oral anticoagulant use (*p* = 0.027), no antiaggregant use (*p* = 0.004), and nondepleted iron stores (*p* = 0.044). No significant associated risk factors for lumbar osteoporosis were detected in this patient group. The univariate analyses of factors associated with osteopenia and osteoporosis are presented in Table 2.

**TABLE 2:** Univariate analysis of factors associated with osteopenia and osteoporosis.

	No osteopenia (*n* = 27)	Osteopenia (*n* = 67)	*p*	No osteoporosis (*n* = 63)	Osteoporosis (*n* = 31)	*p*
Female, *n* (%)	9 (33.3)	46 (68.7)	0.002	32 (50.8)	23 (74.2)	0.030
Disease duration, years, median (range)	1.53 (0.19–16.07)	4.85 (0.07–24.3)	0.032	—	—	—
Cytoreduction, yes/no, *n* (%)	16/11 (59.3/40.7)	53/14 (79.1/20.9)	0.049	—	—	—
ET^#^/non‐ET, *n* (%)	—	—	—	23/40 (36.5/63.5)	19/12 (61.3/38.7)	0.023
Antiaggregant use, yes/no, *n* (%)	—	—	—	52/11 (82.5/17.5)	18/13 (58.1/41.9)	0.011
Current age, years, median (range)	—	—	—	60.1 (22.1–90.8)	66.2 (39.2–88.9)	0.044

*Note:* Only variables with statistically significant associations in univariate analyses are shown. Percentages are calculated within column groups.

^#^Essential thrombocythemia.

The multivariate logistic regression analyses are summarized in Table 3. Female sex remained an independent risk factor for osteopenia, while older age was independently associated with osteoporosis. A BMI > 24.9 emerged as an independent protective factor for femoral osteoporosis. An age cutoff of 59.1 years predicted osteoporosis with 90.3% sensitivity and 44% specificity (AUC: 0.628, CI: 0.515–0.742). A BMI cutoff of 22.4 predicts femoral osteoporosis with 64.3% sensitivity and 96.2% specificity (AUC: 0.834, 95% CI: 0.707–0.961).

**TABLE 3:** Multivariate logistic regression analysis for osteopenia, osteoporosis, and femoral osteoporosis.

Outcome	Variable	Odds ratio	95% CI	*p*
Osteopenia	Female sex	3.70	1.394–9.833	0.009
Osteoporosis	Age (per year increase)	1.04	1.001–1.083	0.044
Femoral osteoporosis	BMI > 24.9	0.088	0.017–0.461	0.004

*Note:* Only univariate associations were put in the logistic regression model.

## 4. Discussion

An increased risk of fractures has been shown in a large population‐based study in MPNs, particularly ET/PV and chronic myeloid leukemia [8]. Supporting this study, another Swedish population–based study showed that the risk of fractures has increased in MPNs, especially in PV and unclassified MPNs, and the risk of fracture is more pronounced in the elderly and in women [9]. While the mechanisms underlying the increased risk of fractures are being investigated, it has also been explored whether secondary osteoporosis develops in this patient group [10].

The absence of a matched control group and the relatively small sample size limit the strength of direct prevalence comparisons with the general population. In our study, both osteopenia and osteoporosis were observed to be more frequent when compared with national data [11]. These findings support the observation that osteopenia and osteoporosis appear to be more frequent in this patient group, particularly among individuals aged over 50 years.

Studies on secondary osteoporosis in terms of ET and PV are scarce in the literature, and secondary osteoporosis is considered speculative [9, 10]. In the study by Farmer et al., 45 PV and ET patients did not show a decrease in bone mineral density compared with a control group matched for age, sex, height, and weight [10]. In contrast to this study, osteoporosis was observed to be significantly more common in ET, in all age groups. In addition, it has been reported that there was no decrease in bone mineral density in 18 patients with MF compared to controls in the literature [11].

It has been stated that salicylates may be safe in terms of osteoporosis, but there is no available data on hydroxyurea and anagrelide. Some researchers suggest that JAK inhibitors may reduce the risk of fractures due to their ability to lower inflammatory markers; however, there is no specific study on this subject [4]. In a recent cross‐sectional study, cytoreductive therapy was associated with a lower prevalence of symptomatic osteoarthritis in patients with chronic MPNs [12]. However, in our cohort, cytoreductive therapy was not independently associated with osteopenia after multivariate adjustment. Similarly, no association was shown between the drugs, such as antiplatelet agents, anticoagulants, and osteoporosis. The relationship between polypharmacy and drug–drug interaction with osteoarthritis/osteoporosis in the MPN patient group has been shown in one study [13]. A limitation of that study was that it did not evaluate polypharmacy.

In previous studies, the distribution of MPN‐10 scores in BCR::ABL1‐negative MPNs showed heterogeneity [14–16], which was found to be generally mild in this study.

In a multinational survey including our country [16], the mean total symptom scores were 23.5, 14.6, and 16.6 in MF, ET, and PV, respectively, whereas in this study, they were 12.2, 15.5, and 14, respectively. However, according to the results of this study, the MPN‐10 score did not play a role in predicting osteopenia/osteoporosis.

The most important limitation of the study is that, because of the retrospective nature of the study, there was no available data on bone turnover markers. Additionally, bone imaging modalities other than DXA, such as peripheral quantitative computed tomography (pQCT), which could provide complementary information on volumetric bone density and bone microarchitecture, were not available. Additionally, referral bias cannot be excluded. Patients with BCR::ABL1‐negative MPNs may experience bone pain as part of their disease symptom burden, which could increase the likelihood of undergoing DXA assessment. Therefore, the observed prevalence of osteopenia and osteoporosis may be influenced by selection bias.

The most important advantage of this study is that it is the first and only study in the literature that evaluates the frequency of both osteopenia and osteoporosis in this patient group and examines their clinical associations in detail. When the study was completed, patients with osteoporosis were informed and referred to relevant specialties for osteoporosis management. It was observed that 11 patients who had never received treatment for osteoporosis were started on treatment (10 patients were prescribed bisphosphonates, and one patient was prescribed denosumab), and one patient who had previously received bisphosphonate therapy was switched to denosumab. Importantly, this study also contributed to the clinical management of patients.

In conclusion, patients with BCR::ABL1‐negative classical MPNs appear to have a higher burden of osteopenia and osteoporosis compared with population‐based data. DXA measurements should be performed, especially in female patients and in patients of advanced age.

## Funding

This research did not receive any specific grant from funding agencies in the public, commercial, or not‐for‐profit sectors.

## Conflicts of Interest

The authors declare no conflicts of interests.

## Data Availability

The data used to support the findings of this study are available from the corresponding author upon request.
